# Body Lice, *Yersinia pestis* Orientalis, and Black Death

**DOI:** 10.3201/eid1605.091280

**Published:** 2010-05

**Authors:** Saravanan Ayyadurai, Florent Sebbane, Didier Raoult, Michel Drancourt

**Affiliations:** Université de la Méditerranée, Marseille, France (S. Ayyadurai, D. Raoult, M. Drancourt); Inserm 1019, Lille, France (F. Sebbane)

**Keywords:** Yersinia pestis, Yersinia pestis biotypes, body lice, black death, plague, bacteria, letter

**To the Editor:** Wild rodent fleas are the most common vectors of *Yersinia pestis*, the plague agent (*1*). The human body louse (*Pediculus humanus*) has been proposed as a probable additional vector during historical epidemics (*2*) because human cases of louse-borne plague have been suspected (*3*) and body louse–borne plague has been demonstrated experimentally with rabbits (*4*). Using rabbits, we tested the ability of the 3 *Y. pestis* main biovars to produce a successful rabbit-louse-rabbit-louse cycle of transmission (*4*).

Two New Zealand White (*Oryctolagus cuniculi*) rabbits were inoculated intravenously with phosphate-buffered saline alone (negative controls) or phosphate-buffered saline containing 10^9^ CFU of *Y. pestis* biotype Nairobi-Rattus Antiqua, biotype 14–47 Medievalis, or biotype 6/69M Orientalis. PCR ensured detection of the virulence factor–encoding plasmids. The rabbits inoculated with biotypes Antiqua, Medievalis, or Orientalis had septicemia of ≈2 × 10^3^ CFU/mL of blood 14 hours postinoculation and died at 20–22 hours, 18–20 hours, or 16–18 hours postinoculation, respectively. In contrast, the negative control rabbits remained healthy for 3 weeks. Five minutes postinoculation, 150 uninfected lice fed for 1 hour on rabbits and took an equivalent blood meal as measured by weight, regardless of the rabbit used. *Y. pestis* was isolated from all 120 randomly tested lice and their feces. Five days postinfection, the death rate of Orientalis-fed lice (95.3%) was significantly higher than that of the control (4%), Antiqua-fed, (78.6%), and Medievalis-fed (74%) (p<0.0001) lice. One third of Orientalis-infected lice remained alive 3 days after the contaminating blood meal.

Lice fed on septicemic rabbits further fed on 2 uninfected rabbits for 1 hour daily for up to 6 days. The rabbits bitten by Orientalis-infected lice had 2.7 × 10^2^ CFU/mL of blood 4 days postinfection and died 1 day later. In contrast, the rabbits bitten by Antiqua-infected or Medievalis-infected lice looked healthy and lacked septicemia 3 weeks after challenge. New groups of 150 uninfected lice fed for 1 hour daily on Orientalis-infected rabbits started to die earlier than did lice fed on Antiqua, Medievalis (1 vs. 2–3 days after blood meal), and uninfected rabbits. Furthermore, 21 days after their first blood meal, lice fed on Orientalis-infected rabbits had a significantly higher death rate (90%) than did control (3%) (p<0.0001), Antiqua-infected (16%), and Medievalis-infected (10%) lice; the latter values were significantly higher than that of the negative controls (p = 0.046). *Y. pestis* could be cultured only from lice and their feces if the lice were fed on rabbits previously bitten by Orientalis-infected lice (Appendix Figure).

Our observation that body lice effectively transmitted *Y. pestis* through a complete cycle of transmission confirms previous experimental (*4*) and field observations of experimental transmission that used body lice collected from plague patients from the same family in the absence of any other ectoparasite (*3*). Transmission of Orientalis but not Antiqua or Medievalis organisms did not result merely from experimental bias because negative controls remained negative, data were duplicated, rabbits exhibited equivalent bacteremia, and lice took equivalent blood meals regardless of biotype.

Our observations shed new light on the Black Death, a medieval epidemic of plague (*5*). Historical records indicate that persons with the Black Death had bubonic plague, indicating an ectoparasite-borne transmission (*1*). *Pulex irritans* fleas were documented in a medieval setting in Viking Greenland (*6*). However, their poor competence (*7*) and the Black Death that swept Russia and Scandinavia are not fully compatible with flea-borne transmission alone. Ten infected lice are sufficient for plague transmission (*4*), and our observation that one third of infected lice remained alive 3 days after infection indicates that an index plague patient carrying as few as 30 body lice could be a source for plague up to 3 days after dying. This figure was highly plausible during the Black Death because body lice currently infest almost 85% of homeless persons, with a mean of 57 lice per person (*8*). Although the role of fleas as vectors of *Y. pestis* from rodents to humans is undisputed, this tabulation sustains the potential role of body lice as an additional vector of plague from human to human during the Black Death (*2*).

Paleomicrobiology suggested that most historical cases in Europe resulted from Orientalis (*5*). This observation challenged the scenario that Antiqua, Medievalis, and Orientalis were responsible for ancient, medieval, and modern plague pandemics, respectively (*9*). The latter scenario had been hypothesized after the biotypes were observed to have a geographic repartition matching that of the hypothetical sources of the 3 historical pandemics (*10*) and was further propagated as dogma without further confirmation.

Our data support an alternative scenario of the historical plague epidemics transmitted by body lice, with Orientalis being the only such louse-borne transmissible biotype. This point justifies studies during ongoing epidemics in cold countries, keeping in mind the need to understand and control re-emerging plague in modern populations exposed to body lice.

## Supplementary Material

Appendix Figure Immunofluorescent detection (polyclonal antibody, original magnification ×100) of *Yersinia pestis* in the feces of body lice during cycles 1 and 2. A) Biotype Antiqua–infected lice feces during cycle 1. B) Biotype Antiqua–infected lice feces during cycle 2. C) Biotype Medievalis–infected lice feces during cycle 1. D) Biotype Medievalis–infected lice feces during cycle 2. E) Biotype Orientalis–infected lice feces during cycle 1. F) Biotype Orientalis–infected lice feces during cycle 2. G) Control lice feces during cycle 1. H) Control lice feces during cycle 2.
