# Supplementary figures and images for: Body Lice, Yersinia pestis Orientalis, and Black Death
Source: Emerg Infect Dis. 2010 May;16(5):892–3. doi: 10.3201/eid1605.091280 (PMC2953993; doi:10.3201/eid1605.091280)

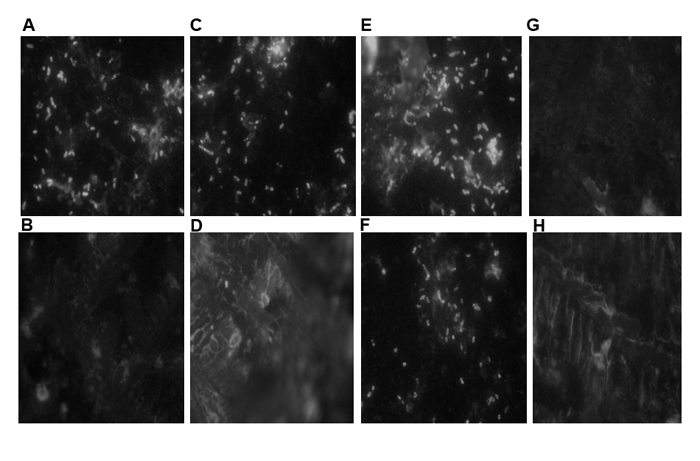

Supplement: Appendix Figure — Immunofluorescent detection (polyclonal antibody, original magnification ×100) of Yersinia pestis in the feces of body lice during cycles 1 and 2. A) Biotype Antiqua–infected lice feces during cycle 1. B) Biotype Antiqua–infected lice feces during cycle 2. C) Biotype Medievalis–infected lice feces during cycle 1. D) Biotype Medievalis–infected lice feces during cycle 2. E) Biotype Orientalis–infected lice feces during cycle 1. F) Biotype Orientalis–infected lice feces during cycle 2. G) Control lice feces during cycle 1. H) Control lice feces during cycle 2. [file 09-1280_appF-s1.gif]
